# Identification of Cytolytic CD161^−^CD56^+^ Regulatory CD8 T Cells in Human Peripheral Blood

**DOI:** 10.1371/journal.pone.0059545

**Published:** 2013-03-19

**Authors:** Dan Hu, Howard L. Weiner, Jerome Ritz

**Affiliations:** 1 Center for Neurologic Diseases, Brigham and Women’s Hospital, Boston, Massachusetts, United States of America; 2 Division of Hematologic Malignancies, Cancer Vaccine Center, Dana-Farber Cancer Institute, Boston, Massachusetts, United States of America; 3 Harvard Medical School, Boston, Massachusetts, United States of America; University of Florence, Italy

## Abstract

We previously developed methods for establishing CD8 regulatory T cell (Treg) clones from normal human peripheral blood and demonstrated that these clones were capable of killing T cell receptor (TCR)-activated autologous CD4 T cells. Based on phenotypic and functional characterization of the CD8 Treg clones, we have identified a corresponding population of endogenous CD8 Treg in normal human peripheral blood. These cells appear morphologically as large lymphocytes with abundant cytoplasm and have the following unique phenotype: CD3^+^CD8^+^CD161^−^CD56^+^. The majority of CD8 Treg express CD45RA and CD62L with low or negative expression of CD45RO, CD25, CD27, CD28 and CCR7. The expression of CD94 and NKG2a on CD8 Treg was elevated compared to conventional CD8 T cells. Following in vitro activation, this T cell subset is capable of killing TCR-activated CD4 T cells. These studies identify an endogenous CD8 Treg population in humans and it will now be possible to characterize these cells in a variety of clinical conditions.

## Introduction

Immune responses are controlled by various populations of T cells with regulatory function. These regulatory T cells (Treg) suppress activated immune cells thereby maintaining immune system homeostasis and self-tolerance [1], [2], [3], [4], [5], [6], [7]. Although the phenotype and function of CD4 Treg have been characterized in great detail, CD8 Treg have not been well characterized. In murine models, CD8 Treg contribute to resistance to experimental allergic encephalomyelitis (EAE), a model for human multiple sclerosis [8], [9]. Adoptive transfer of CD122^+^CD8^+^ T cells prevents development of abnormal T cells in CD122-deficient mice [10]. More recent studies have shown that CD8 Treg suppress pathogenic autoreactive CD4 T cells via a Qa-1-restricted pathway [11]. Genetic disruption of the inhibitory interaction between these CD8 T cells and their target Qa-1-expressing CD4 T cells results in increased susceptibility to EAE [11], [12] and development of a lethal systemic-lupus-erythematosus-like autoimmune disease [13]. CD8 Treg have also been identified in patients with multiple sclerosis [14], ovarian carcinoma [15] and HIV-infection [16]. Several phenotypes of CD8 Treg have previously been reported including; CD8^+^CD103^+^ [17], CD8^+^CD25^+^CD28^+^Foxp3^+^ [18], CD8^+^CD28^−^Foxp3^+^ [19], CD8^+^CD122^+^ [10], and CD8^+^CCR7^+^CD45RO^+^IL10^+^ [15]. It is not clear whether different CD8 Treg subsets represent independent populations or whether they reflect different characteristics of a single population. However, one consistent functional feature of CD8 Treg is that these cells act primarily through suppression of activated CD4 T cells [10], [11], [20], [21]. Moreover, almost all studies on CD8 Treg have been conducted in murine models and few studies have focused on CD8 Treg in humans.

Our limited understanding of CD8 Treg populations and the inherent uncertainty of extrapolating from mouse models to humans led us to develop a novel protocol to establish stable CD8 T cell clones with auto-regulatory activity from normal human peripheral blood [22]. CD8 Treg clones effectively suppressed activated CD4 T cells and expressed a variety of TCR Vβ chains, indicating that the CD8 Treg population in humans is polyclonal. Suppression by CD8 Treg clones was cell contact-dependent, involved CD11a/CD18 (LFA-1) and CD8 surface antigens and resulted in lysis of CD4^+^ target T cells. Moreover, suppression by CD8 Treg was independent of the antigen-specificity of CD4^+^ target T cells and HLA compatibility between effector and target cells [22]. CD8 Treg clones were CD8αβ^+^TCRαβ^+^TCRγδ^−^TCRVα24^−^TCRVβ11^−^ and did not expressed significant levels of CD28, CD103, CD122, CCR7 and IL-10. Unlike CD4 Treg, which are largely defined by the expression of Foxp3 [1], [23], [24], levels of Foxp3 expression in CD8 Treg clones were not correlated with their suppressive activity [22]. The lack of CD28, CD103, CD122, CXCR4 and CCR7 expression and dissociation of Foxp3 expression from suppressive activity indicate that CD8 Treg clones are different from the CD103^+^, CD28^−^Foxp3^+^, CD25^+^CD28^+^Foxp3^+^, CD122^+^ and CCR7^+^CD45RO^+^IL10^+^ CD8 Treg subsets previously reported. Interestingly, CD8 Treg clones frequently expressed CD56 and rarely expressed CD161 [22], even though CD56 and CD161 are often co-expressed on NK and NKT cells [25], [26].

The establishment of stable human CD8 Treg clones has provided us with a consistent experimental system to characterize human CD8 Treg in vitro and to identify phenotypic characteristics that can be used to define the corresponding endogenous population in vivo. In the present study, we describe a population of CD3^+^CD8αβ^+^CD161^−^CD56^+^Vα24^−^ T cells in normal human peripheral blood that function as CD8 Treg. Like CD8 Treg clones, these CD8 Treg kill TCR-activated CD4 T cells independent of the antigen-specificity of CD4 target T cells and HLA compatibility between effector and target cells.

## Results

### Presence of CD3^+^CD161^−^CD56^+^ CD8 T Cell Subset in Normal PBMC

**Figure 1:**
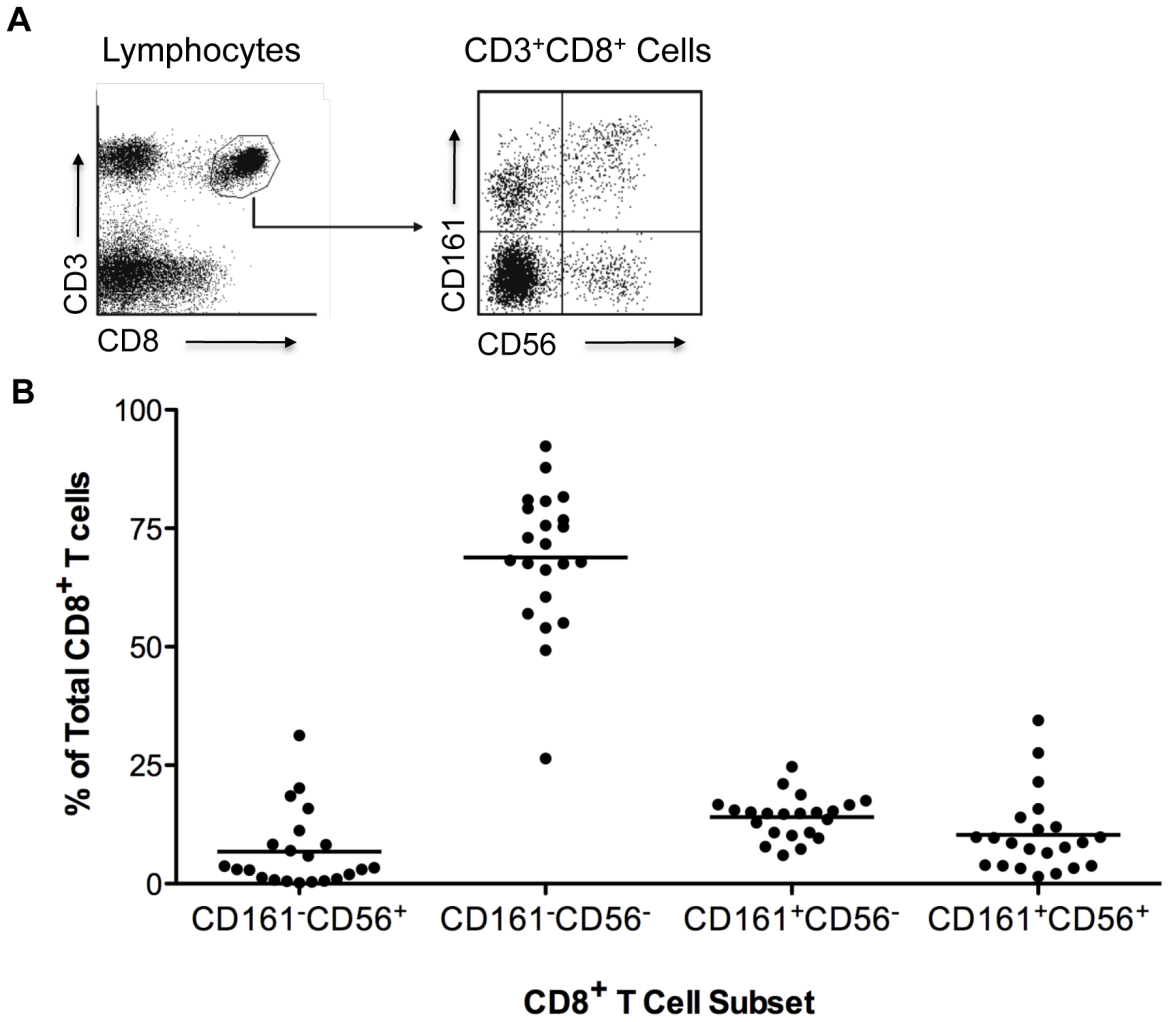
CD161^−^CD56^+^ CD8 T cell subset in normal PBMC. (A) Existence of CD161^−^CD56^+^ CD8 T cell subset in normal PBMC. Flow cytometric analysis of cell surface expression of CD3, CD8, CD161 and CD56 on PBMC from healthy donors. The left plot was gated on lymphocytes and the right plot on CD8 T cells. Plots are representative of PBMC from 22 healthy donors. (B) Percentage of CD161^−^CD56^+^ CD8 T cells in total CD8 T cells. Each dot represents one individual (n = 22). Horizontal bars indicate the mean. Cell subset gating was as shown in part (A) right plot.

CD56 and CD161 are natural killer (NK) cell and natural killer T (NKT) cell surface markers [1], [22], [23], [24], [25], [26], which are often co-expressed. In contrast, Vα24 and Vβ11 are characteristic cell surface markers for NKT cells [27], [28], [29]. CD8 Treg clones expressed CD56, but were Vα24^−^Vβ11^−^ and generally did not express CD161. This characteristic phenotype distinguished the CD8 Treg clones from NKT cells and the dissociated expression of CD56 and CD161 in CD8 Treg clones prompted us to examine whether CD8 T cells expressing CD56 but not CD161 might represent a unique in vivo population corresponding to the CD8 Treg clones. To examine whether a CD161^−^CD56^+^ CD8 T cell subset exists, we analyzed PBMC from 22 anonymous healthy donors that had been stained with fluorochrome-conjugated anti-CD3, CD8α, CD56 and CD161 monoclonal antibodies. The dominant subset of CD8 T cells was CD56^−^ and CD161^−^, representing conventional CD8 T cells (CD8 Tcon). CD3^+^CD8^+^CD161^+^CD56^+^ and CD3^+^CD8^+^CD161^+^CD56^−^ cells, representing NKT cells were also present in all individuals. However, there was a distinct population of CD8 T cells that expressed CD56 but not CD161 in each individual (Figure 1A). The percentage of this population varied widely from 0.2% to 31.3% of all CD8^+^ T cells with a median of 3.2% and mean of 6.8%. Seventeen of the 22 donors had less than 10% CD8 T cells that were CD161^−^CD56^+^ (Figure 1B). Since these were anonymous donor samples, we were not able to examine whether the variable frequency of CD161^−^CD56^+^ CD8 T cells correlated with gender and/or age. However, these results demonstrated that CD3^+^CD8^+^CD161^−^CD56^+^ cells are a distinct phenotypic CD8 T cell subset presented in human peripheral blood in healthy individuals.

### Cell Surface Antigens Expressed by CD161^−^CD56^+^ CD8 T Cells

**Figure 2:**
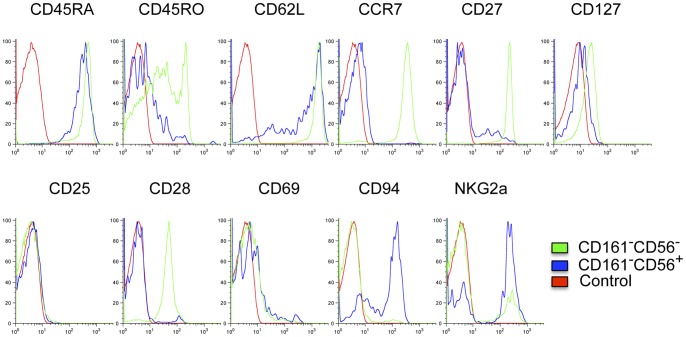
Distinct cell surface antigen expression profile of CD161^−^CD56^+^ CD8 T cells. **** PBMC isolated from healthy donors (n = 5) were stained and analyzed as described in Materials and Methods. Plots shown are from one representative individual. Conventional CD8 T cells (CD161^−^CD56^−^) are compared to CD8 Treg (CD161^−^CD56^+^) and Control (non-stained lymphocytes).

**Figure 3:**
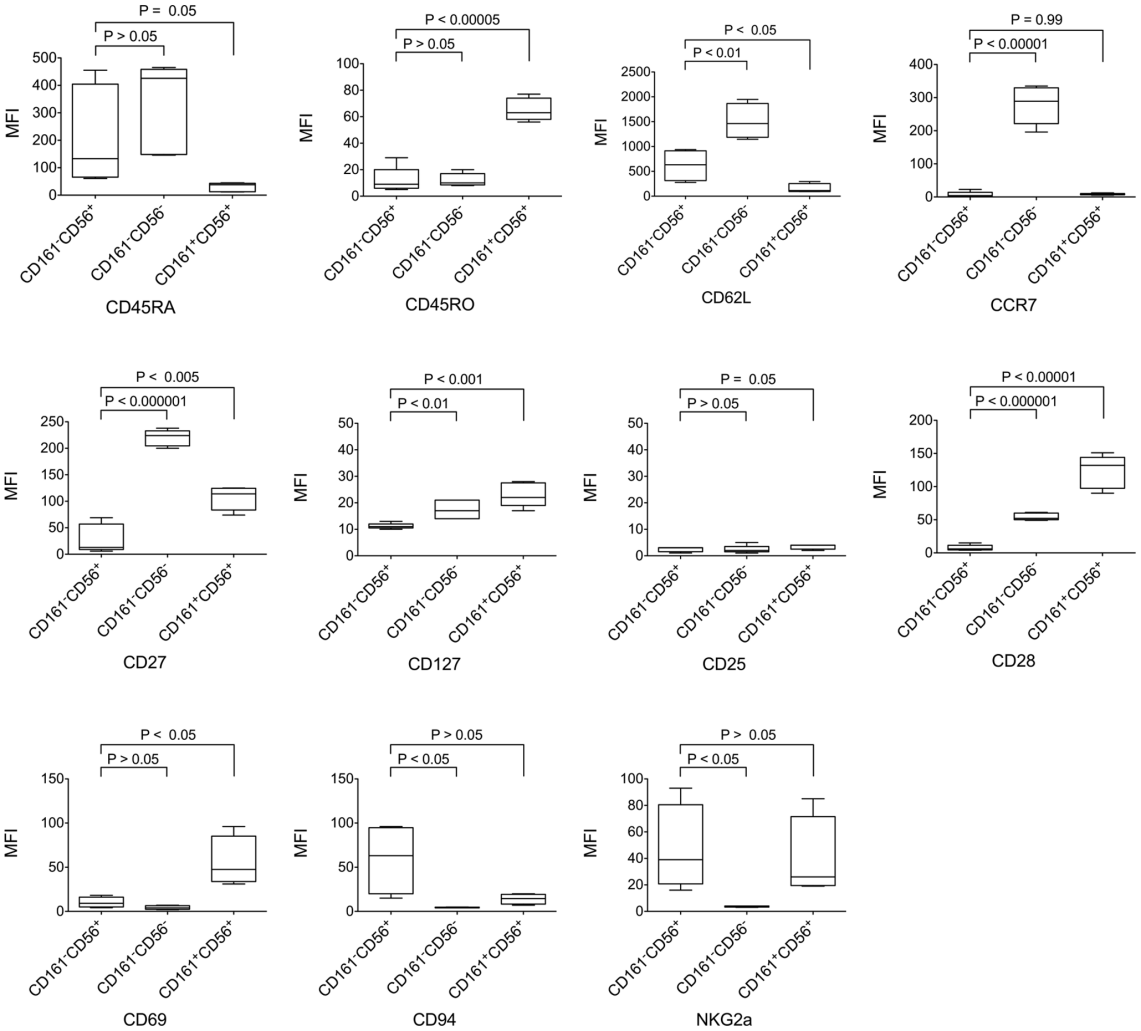
Cell surface antigens expressed by CD161^−^CD56^+^ CD8 T cells. PBMC isolated from healthy donors were stained and analyzed as described in Materials and Methods. Intensity of cell surface expression of various markers shown in each graph is compared for different CD3^+^CD8^+^ subsets: CD8 Treg (CD161^−^CD56^+^); conventional CD8 T cells (CD161^−^CD56^−^); and CD8 NKT cells (CD161^+^CD56^+^). Mean and standard deviation are shown (n = 5).

Further characterization of cell surface antigens expressed by CD3^+^CD8^+^CD161^−^CD56^+^ cells demonstrated distinct patterns of expression when compared with conventional CD3^+^CD8^+^CD161^−^CD56^−^ T cells (CD8 Tcon) (Figure 2 and 3) and CD3^+^CD8^+^CD161^+^CD56^+^ NKT cells (Figure 3). The large majority of CD3^+^CD8^+^CD161^−^CD56^+^ cells express the naïve cell surface marker CD45RA and are negative for memory/effector cell surface marker CD45RO. In contrast, CD3^+^CD8^+^CD161^+^CD56^+^ NKT cells were consistently CD45RA^−^ and CD45RO^+^. Both CD8 Tcon and CD8 Treg subsets expressed CD62L (Figure 2 and 3), but the level of CD62L expression level was significantly lower in CD8 Treg compared to CD8 Tcon (Figure 3). CD27, a member of the tumor necrosis factor receptor (TNFR) superfamily, is expressed on the majority of CD4 and CD8 T cells, especially naïve T cells [30]. In our analysis, CD27 was highly expressed on CD8 Tcon, however, most CD8 Treg cells were CD27 negative or low. While the coordinate expression of CD45RA^+^CD45RO^−^CD62L^+^ suggested that CD8 Treg have a naïve phenotype, these cells were CCR7^−^CD27^low/−^CD28^−^, suggesting that they were likely antigen-experienced. CD8 Treg were also CD25 and CD69 negative, and expressed significantly higher levels of CD94 and NKG2a and lower levels of CD127 compared with CD8 Tcon. Unlike the ambiguous cell surface antigen expression pattern of CD8 Treg, CD3^+^CD8^+^CD161^+^CD56^+^ NKT cells displayed the typical phenotype of effector cells. NKT cells were CD45RA^−^CD45RO^+^CD62L^−^CCR7^−^CD27^low^CD127^+^CD28^hi^CD69^+^. Thus, CD8 Treg express a unique pattern of cell surface antigens (CD45RA^+^CD45RO^−^CD62L^+^CCR7^−^CD27^low/−^CD25^−^CD28^−^), which is distinct from both conventional CD8 T cells and NKT cells.

### CD161^−^CD56^+^ CD8 T Cells Display Abundant Cytoplasm

**Figure 4:**
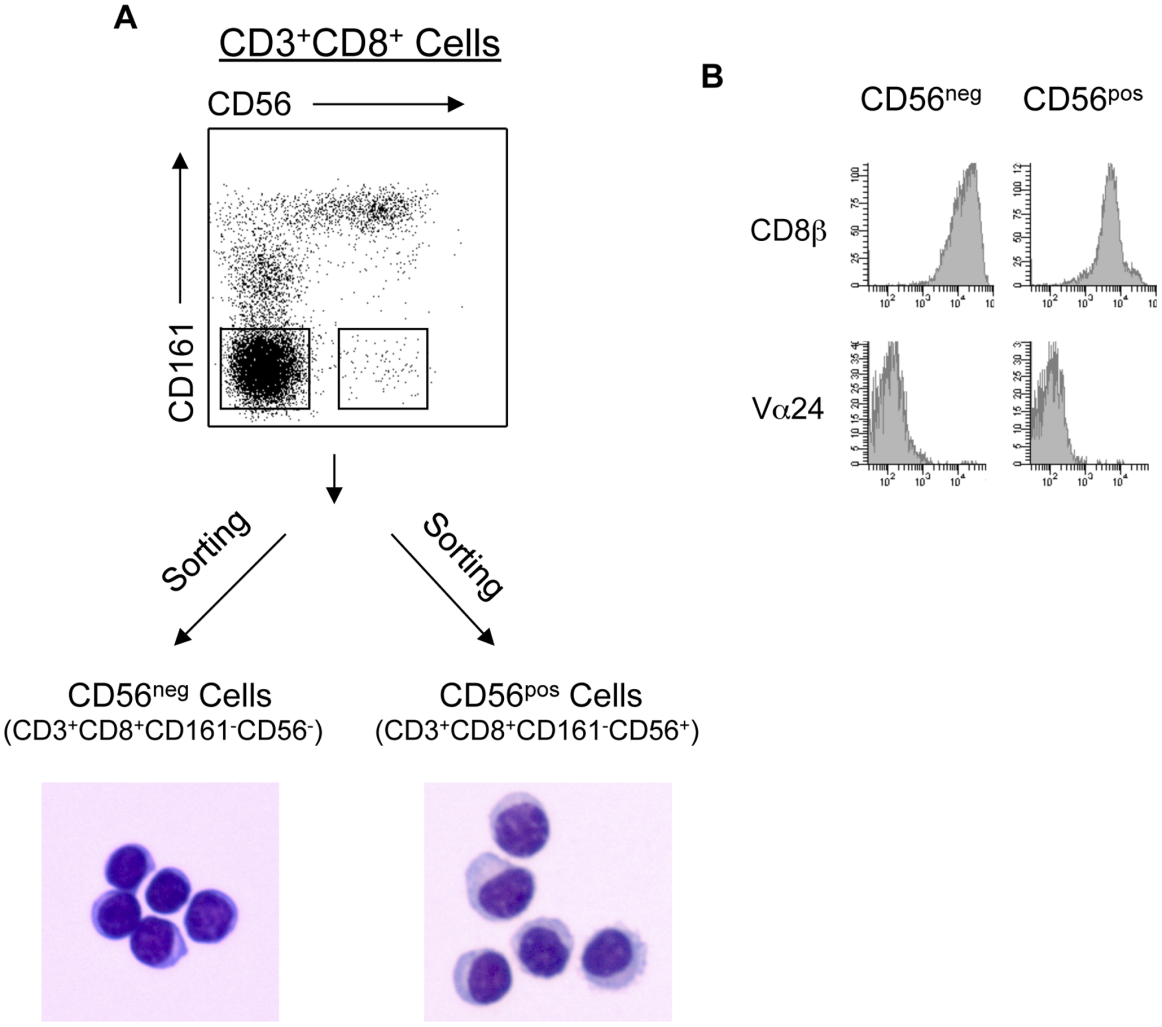
CD161^−^CD56^+^ CD8 Treg are CD8αβ^+^Vα24^−^ cells with abundant cytoplasm. (A) Morphology of CD161^−^CD56^+^ CD8 Treg. PBMC were stained as described in Materials and Methods. CD56^pos^ (CD3^+^CD8^+^CD161^−^CD56^+^) CD8 Treg and CD56^neg^ (CD3^+^CD8^+^CD161^−^CD56^−^) CD8 Tcon were purified by cell sorting. Isolated CD56^pos^ and CD56^neg^ cells were stained with Wright-Giemsa stain and visualized using a bright-field microscope. Photos are from one representative experiment of six. (B) Cell surface expression analysis of CD8**β** and V**α**24 on CD56^pos^ and CD56^neg^ cells. CD56^pos^ (CD8 Treg) and CD56^neg^ (CD8 Tcon) were purified by fluorescence-activated cell sorting. Both subsets were CD161 negative. Sorted cells were analyzed for expressions of CD8**β** and V**α**24 as described in Materials and Methods. Data are from one of four representative healthy donors.

To further characterize this population, CD161^−^CD56^+^ CD8 Treg cells (CD56^pos^) from normal human PBMC were purified by fluorescence-activated cell sorting (FACS). CD3^+^CD8^+^CD161^−^CD56^−^ (CD56^neg^) cells were collected simultaneously as conventional CD8 T cell controls. CD56^neg^ cells displayed typical morphology of mature lymphocytes with a round nucleus and little cytoplasm. In contrast, CD3^+^CD8^+^CD161^−^CD56^+^ (CD56^pos^) cells had abundant cytoplasm with occasional granules and irregular nuclei (Figure 4A). These morphologic features are typical of activated T cells and NK cells. Both CD56^pos^ (CD8 Treg) and CD56^neg^ cells (CD8 Tcon) were CD8β^+^TCRVα24^−^ (Figure 4B). Being CD8β^+^TCRVα24^−^CD161^−^ indicates that the CD56^pos^ T cell population, like the CD56^neg^ T cell population, did not include NKT cells [30].

### CD8^+^CD161^-^CD56^+^ T Cells Maintain Low Level Expression of CD27 after Activation

**Figure 5:**
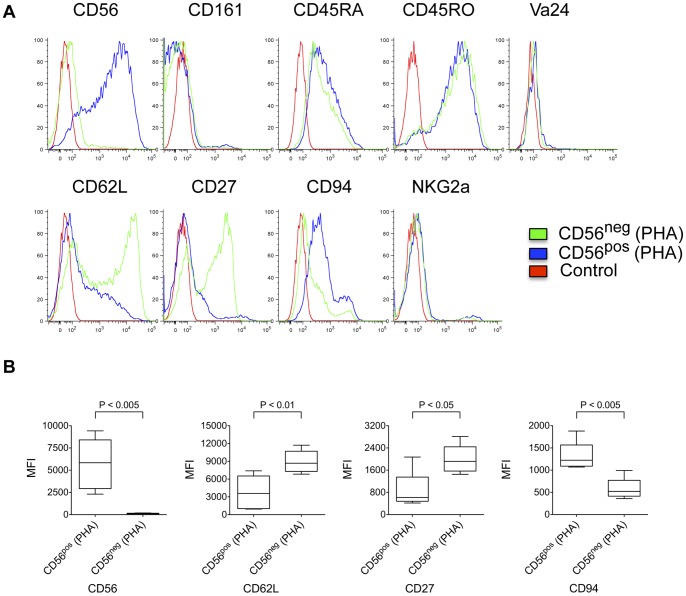
Cell surface phenotype of PHA-activated CD8 Treg. (A) Representative histograms for cell surface antigen expression on PHA activated CD8 Treg (CD56^pos^) and CD8 Tcon (CD56^neg^). CD56^pos^ and CD56^neg^ cells isolated from PBMC were stimulated with PHA and cultured in the presence of feeder cells for 8 days before being analyzed for cell surface marker expression as described in Materials and Methods. Data shown are from one of 5 representative healthy donors. (B) Activated CD56^pos^ cells maintained high expression of CD56 and CD94 and lower expression of CD62L and CD27. CD8 Treg (CD56^pos^) and CD8 Tcon (CD56^neg^) cells isolated from PBMC were stimulated with PHA and cultured in the presence of feeder cells for 9 to 16 days before being analyzed for intensity of expression of CD56, CD62L, CD27 and CD94 cell surface antigens. *p*-value was calculated with unpaired two-tailed Student’s t-test. Mean and standard deviation are shown (n = 5).

To activate and expand CD8 Treg, we stimulated FACS-purified CD8^+^CD161^−^CD56^+^ cells with phytohemagglutinin (PHA) in the presence of feeder cells. CD8 Treg (CD56^pos^) cells proliferated as robustly as CD8 Tcon (CD56^neg^) cells cultured in parallel. After activation, CD56^pos^ cells remained CD161^−^Vα24^−^, and maintained higher expression of CD94 and lower expression of CD62L compared with expanded CD56^neg^ cells (Figure 5A and 5B). After in vitro expansion, both CD56^pos^ cells and CD56^neg^ populations lost expression of CD45RA and became strongly CD45RO positive. It has been reported that activation of T cells via TCR/CD3 induces high surface expression of CD27 [30]. However, the expression of CD27 in CD8 Treg (CD56^pos^) cells remained low (Figure 5A and 5B).

### Lysis of Activated Allogeneic and Autologous CD4^+^ T Cells by CD8^+^CD161^−^CD56^+^ T cells

**Figure 6:**
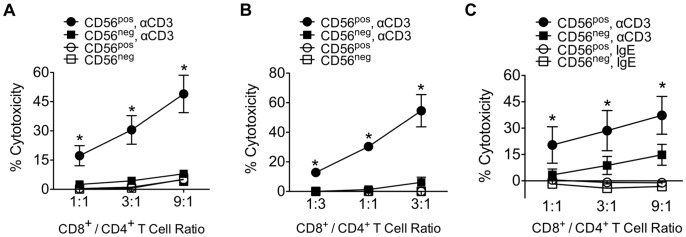
Activated CD56^pos^ cells induce cell death in TCR-activated allogeneic and autologous CD4 T cells. (A) Suppression of activated allogeneic clonal CD4 T cells by CD8 Treg (CD56^pos^) cells. CD56^pos^ and CD56^neg^ cells isolated from healthy donors were stimulated with PHA and expanded for 7–9 days. Suppression assays were performed as described in Materials and Methods. (CD56^pos^, αCD3), the suppressive activity of CD8 Treg towards CD3-activated S2B5 cells; (CD56^neg^, αCD3), the suppressive activity of CD8 Tcon towards CD3-activated S2B5 cells; (CD56^pos^), the suppressive activity of CD8 Treg towards non-activated S2B5 cells; (CD56^neg^), the suppressive activity of CD8 Tcon towards non-activated S2B5 cells. *p<0.05. Results are pooled from three healthy donors (n = 3). *p* values were calculated with unpaired two-tailed Student’s t-test compared to (CD56^neg^, αCD3). Mean and standard deviation are shown. (B) Activated CD8 Treg induce lysis of TCR-activated autologous polyclonal CD4 T cells. Isolated CD8 Treg (CD56^pos^) and CD8 Tcon (CD56^neg^) were stimulated with PHA and expanded for 8–15 days. Isolated autologous polyclonal CD4 T cells were stimulated with PHA and expanded for 8 days. Suppression assays were performed as described in Materials and Methods. (CD56^pos^, αCD3), the suppressive activity of CD8 Treg towards pre-activated autologous CD4 T cells; (CD56^neg^, αCD3), the suppressive activity of CD8 Tcon towards pre-activated autologous CD4 T cells; (CD56^pos^), the suppressive activity of CD8 Treg towards non-activated autologous CD4 T cells; (CD56^neg^), the suppressive activity of CD8 Tcon towards non-activated autologous CD4 T cells. *p<0.05. Results are pooled from two independent sorting experiments. *P* values were calculated with unpaired two-tailed Student’s t-test compared to (CD56^neg^, αCD3). Mean and standard deviation are shown. (C) Activated CD8 Treg induce lysis of TCR-activated allogeneic polyclonal CD4 T cells. Isolated CD56^pos^ and CD56^neg^ cells were stimulated with PHA and expanded for 8–15 days. Isolated allogeneic polyclonal CD4 T cells were stimulated with PHA and expanded for 8–10 days. Suppression assays were performed as described in Materials and Methods. (CD56^pos^, αCD3), the suppressive activity of CD8 Treg towards pre-activated allogeneic polyclonal CD4 T cells; (CD56^neg^, αCD3), the suppressive activity of CD8 Tcon towards pre-activated allogeneic polyclonal CD4 T cells; (CD56^pos^, IgE), the suppressive activity of CD8 Treg towards isotype control mouse IgE-treated allogeneic polyclonal CD4 T cells; (CD56^neg^, IgE) the suppressive activity of CD8 Tcon towards isotype control mouse IgE-treated allogeneic polyclonal CD4 T cells. *p<0.05. Results are pooled from six healthy donors (n = 6). *p*-values were calculated with unpaired two-tailed Student’s t-test compared to (CD56^neg^, αCD3). Mean and standard deviation are shown.

We have previously shown that human CD8 Treg-mediated suppression requires TCR activation of CD4^+^ target T cells but is independent of the antigen-specificity of CD4^+^ target T cells and HLA compatibility between effector and target cells [22]. To determine whether polyclonal CD8 Treg (CD56^pos^) cells had similar functional activity, CD8^+^CD161^−^CD56^+^ and CD8^+^CD161^−^CD56^−^ T cells were FACS-purified, expanded in vitro for 7–10 days and tested for cytolytic activity against an allogeneic Epstein-Barr virus (EBV)-specific CD4 T cell clone (S2B5) we previously established and used as target cells for CD8 Treg clones [22]. Activated CD8 Treg (CD56^pos^) lysed the CD4^+^ target cell clone (S2B5), but only after target cells were sensitized through TCR activation with anti-CD3 monoclonal antibody. In contrast, activated CD8 Tcon (CD56^neg^ ) cells had no specific lytic activity in this assay, either with or without TCR activation of the CD4^+^ target cells (Figure 6A). To further examine their functional activity, CD8 Treg (CD56^pos^), CD8 Tcon (CD56^neg^), and autologous polyclonal CD4 T cells were purified by flow cytometric cell sorting. Sorted CD4 T cells, CD8 Treg and CD8 Tcon were expanded in the presence of PHA and feeder cells. Activated CD8 Treg (CD56^pos^) cells effectively killed anti-CD3 antibody-sensitized autologous polyclonal CD4 T cells, but not non-sensitized cells. In contrast, activated CD8 Tcon (CD56^neg^) cells did not display significant cytotoxicity towards the autologous polyclonal CD4 T cells with or without TCR activation of the CD4^+^ target cells (Figure 6B). In addition, we also isolated allogeneic polyclonal CD4 T cells from PBMC and expanded them in vitro with PHA stimulation for use as target cells. Activated CD8 Treg (CD56^pos^) cells but not CD8 Tcon (CD56^neg^) lysed allogeneic polyclonal CD4 T cells after target cells were sensitized through TCR activation with anti-CD3 (Figure 6C). Thus, like the CD8 Treg clones, activated CD8^+^CD161^−^CD56^+^ T cells effectively killed TCR-activated CD4 T cells regardless of the antigen-specificity of CD4^+^ target T cells and HLA compatibility between effector and target cells. Taken together, these results suggest that the CD8^+^CD161^−^CD56^+^ T cell subset in normal PBMC corresponds to an endogenous population of CD8 Treg.

## Discussion

In previous studies we established a panel of human CD8 Treg clones that selectively suppress activated CD4 T cells in vitro [22]. Here we sought to identify and characterize a population of CD8 T cells in normal human peripheral blood that had similar functional activity. Based on phenotypic and functional characterization of the CD8 Treg clones, we identified a population of CD8αβ^+^CD161^−^CD56^+^ T cells in human peripheral blood that that suppresses activated CD4 T cells and therefore functions as a regulatory T cell subset.

The selection of CD3, CD8, CD56 and CD161 as potential positive and negative markers of peripheral CD8 Treg was based on the detailed phenotypic characterization of CD8 Treg and non-Treg clones we had previously established. Since both sets of clones were established in a similar method and differed primarily by their ability to suppress autologous activated CD4 T cells, we reasoned that the phenotypic comparison of these clones would identify surface markers that could distinguish CD8 Treg from conventional CD8 T cells in normal peripheral blood. When this marker set was subsequently tested in 22 healthy individuals, each donor was found to have a distinct population of CD8^+^CD161^−^CD56^+^ T cells. Morphologically, these cells have moderate amounts of cytoplasm with occasional granules. Overall, cells with this phenotype represented a median of 3.2% of CD8 T cells. Although it is likely that this population remains heterogeneous and contains cells with other functional characteristics, it appears that this relatively small CD8 T cell subset is present in all normal individuals.

To determine whether CD3^+^CD8^+^CD161^−^CD56^+^ cells display similar suppressive activity as CD8 Treg clones, cells with these markers were purified from normal PBMC by flow cytometric cell sorting, stimulated non-specifically with PHA and expanded in vitro. Previous experiments had shown that CD8 Treg clones maintained suppressive activity during prolonged periods of expansion in vitro in similar conditions, and this allowed us to obtain relatively large numbers of cells needed for functional experiments [22]. Following expansion in vitro, CD3^+^CD8^+^CD161^−^CD56^+^ cells continued to express CD3, CD8 and CD56 and did not acquire CD161 expression. There were some changes in cell surface markers as these cells converted from a naïve phenotype (CD45RA^+^CD45RO^−^) to an effector/memory phenotype (CD45RA^−^CD45RO^+^). Most importantly, these expanded cells exhibited functional activity that was identical to the CD8 Treg clones previously established. When tested against an allogeneic EBV-peptide specific CD4 T cell clone (S2B5) and polyclonal autologous and allogeneic CD4 T cells, expanded CD8 Treg were able to lyse target CD4 T cells but this was only evident after activation of CD4 target cells with anti-CD3 monoclonal antibody. Conventional CD8 T cells expanded under identical conditions were not able to kill CD4 target cells, with or without anti-CD3 activation. These experiments do not address the question of whether CD3^+^CD8^+^CD161^−^CD56^+^ cells display CD8 Treg suppressive activity in the absence of activation in vitro. In preliminary experiments, freshly isolated CD3^+^CD8^+^CD161^−^CD56^+^ cells did not exhibit suppressive activity in vitro without subsequent activation and expansion in vitro. CD4 Treg do not require further activation in vitro to exhibit suppressive functions but CD8 Treg may require activation before suppressive activity is acquired. Further experiments to address this important functional comparison with CD4 Treg are necessary to define the cellular requirements for CD8 Treg activity in vivo.

We used cell surface expression of CD56 without concomitant expression of CD161 as the primary identifying feature of this novel CD8 T cell subset. CD56 is a cell surface marker characteristic of all human NK cells and 90% of NK cells also express CD161 [31]. Conventional NK cells do not express CD3 and TCR but some of these cells express CD8αα homodimers [32]. In mice, NKT cells were described as a subset of T cells in thymus that expressed NK-associated cell surface antigen NK1.1 (CD161 in human) [33]. In humans, NKT cells are a heterogeneous group of cells that possess properties of both T cells and NK cells [34], [35]. NKT cells characteristically express CD3 and CD161; most NKT cells express CD4 and few express CD8. Only some NKT cells express CD56 and expression of CD56 is therefore not a distinguishing marker for these cells. In contrast to conventional T cells, NKT cells specifically recognize lipid antigens presented by CD1d molecules. NKT cells typically produce IFN-γ, IL-4 and other cytokines upon activation and are not directly cytolytic. Classical NKT cells (also termed invariant NKT), which constitute more than 80% of NKT cells, express a canonical Vα14-Jα18 α chain with a variable Vβ8, −7, or −2 β chain in mouse or Vα24-Jα18/Vβ11 in human. Non-classical NKT cells in the mouse express a variety of rare but recurrent, CD1d-reactive Vα3.2-Jα9/Vβ8, Vα8/Vβ8, and other TCRs [28], [29]. In contrast to both conventional NK and NKT cells, the CD8 Treg we identified in human peripheral blood express CD3, CD8αβ, TCRαβ and CD56 but not CD161, Vα24 or Vβ11 (data not shown). CD8 Treg are highly lytic towards activated CD4 T cells regardless of HLA compatibility. Finally, CD8 Treg do not secrete high levels of IL-4 upon CD3 activation though the production of IFN-γ is greatly enhanced [22]. Taken together, these results indicate that the CD3^+^CD8αβ^+^TCRαβ^+^CD161^−^CD56^+^Vα24^−^ Treg subset we identified in normal peripheral blood is phenotypically and functionally distinct from both NK and NKT cells.

Further studies demonstrated that CD8 Treg in peripheral blood displayed an unusual pattern of cell surface antigens. Typically, these cells were CD45RA^+^ CD45RO^−^ CD62L^+^ CCR7^−^ CD27^low/−^ CD25^−^ CD28^−^ CD69^−^CD127^low^. Conventional naïve CD8 T cells are usually CD45RA^+^ CD45RO^−^ CD62L^+^ CCR7^+^ CD27^+^ CD25^−^ CD28^+^ CD69^−^ CD127^+^, while conventional effector/memory CD8 T cells are generally CD45RA^−^ CD45RO^+^ CD62L^−^ CCR7^−^. Although CD62L and CCR7 are retained on central memory T cells, these cells are CD45RA^−^CD45RO^+^ [36]. Clearly, this new T cell subset cannot be readily categorized either as conventional naïve T cells or effector/memory T cells. However, unusual expression of these cell surface antigens has been previously reported. For example, it was reported that a new subset of CD8 T cells that are CD45RA^hi^ CD27^hi^ CD127^low^ CD28^low^ HLA-DR^hi^ might represent naive T cells that have recently received homeostatic signals [37], while expression of CD45RA on CCR7^−^ CD8 T cells during viral infections may be a marker for terminally differentiated cells [38]. Further studies are needed to determine whether CD3^+^ CD8^+^ CD161^−^ CD56^+^ cells are naïve or antigen-experienced.

The low or negative expression of CD27 on CD8 Treg cells may be functionally related. CD27 is a member of the TNF-receptor superfamily that is expressed on early thymocytes and naïve T cells. All CD4^+^CD45RA^+^CD45RO^−^ T cells [30] and most NK cells express CD27 [39]. CD70, a unique ligand for CD27 is transiently expressed on activated T cells, B cells and dendritic cells. The CD27 receptor activates the NF-κB and JNK pathways [40] and plays a critical role in B cell activation and differentiation. On conventional T cells, CD27 expression is increased after activation, but is subsequently downregulated as T cells undergo proliferation and acquire effector cell function. An inverse correlation between expression of CD27 and cytotoxic effector function has been reported in both CD8 T cells and NK cells [41], [42]. Our analysis of conventional naïve CD8 T cells confirmed that these cells express CD27 and this was maintained during short term activation in vitro. In contrast, very few freshly isolated CD8 Treg expressed CD27 even though more than 90% of these cells are CD45RA^+^CD45RO^−^. Moreover, CD27 expression on CD8 Treg after PHA activation remained significantly lower than on conventional CD8 T cells. These observations suggest that CD8 Treg may be intrinsically primed toward cytolytic function.

Having identified a phenotypically and functionally distinct population of human CD8 Treg in normal peripheral blood, further studies are needed to define the role of these cells in the regulation of T cell responses and the molecular mechanisms that mediate their function. The ability of CD8 Treg to selectively lyse activated CD4 T cells appears to be a distinct functional program that is not shared by other cytolytic cells including NK cells and conventional cytolytic CD8 T cells. Although these cells express CD8 and TCR, the specificity of these cells is not restricted by expression of identical MHC class I molecules on the target cell surface. Thus the mechanisms used by these cells to selectively recognize and interact with activated CD4^+^ T cells have not been defined. Identification of these interacting structures will be needed to establish the mechanisms by which these cells can regulate immune responses in vivo. Similarly, the derivation of these cells and their relationship to conventional CD8 T cells is also an important area for further investigation. However, the identification of a distinct population of CD8 T cells with the ability to selectively regulate activated CD4 T cells is a critical step in the functional characterization of these cells and will also allow the characterization of these cells in different clinical conditions.

## Materials and Methods

### Antibodies

Monoclonal antibodies (mAb) for CD3, CD8α, CD8β, CD25, CD56, CD62L, CD69, CD94, CD161, Vα24, Vb11 and NKG2a cell surface staining were obtained from Beckman Coulter (Brea, CA). Additional mAbs for CD3, CD8α, CD27, CD28, CD45RA, CD45RO, CD56, CD62L, CD94, CD127 and CD161 were purchased from Biolegend (San Diego, CA). Mouse anti-human CD3 IgE mAb (RDI-M1654clb) was purchased from Research Diagnostics Inc (Flanders, NJ). Purified mouse IgE mAb was purchased from Biolegend (San Diego, CA).

### Cells and Culture

Normal PBMC were isolated from donor leukopack samples provided by the Dana-Farber Cancer Institute Blood Donor Center after Ficoll-Paque (Amersham Pharmacia Biotech) separation. Normal PBMC were also isolated from blood samples from healthy subjects after written consent, which was approved by the institutional review board of the Brigham and Women’s hospital. EBV-transformed B cell lines (LCL) were maintained in RPMI 1640 (Mediatech, Inc) supplemented with 10% heat-inactivated FBS, 2 mM glutamine, 1 mM sodium pyruvate, 10 mM Hepes, 50 I U/ml penicillin and 50 μg/ml streptomycin. T cell clones or lines were maintained and expanded via stimulation with PHA. Briefly, T cells were first stimulated with 1.2 μg/ml PHA (PHA-L, Sigma) in Iscove’s Modified Dulbecco’s Medium (IMDM-Invitrogen) supplemented with 10% heat-inactivated human AB serum (FBS for S2B5 cells), 100 u/ml recombinant human IL-2 (rIL-2), 50 I.U/ml penicillin and 50 μg/ml streptomycin (complete IMDM) in the presence of irradiated PBMC (3,500 cGy, 1×10^6^ or 3×10^6^ cells/well) and LCL (6,000 cGy, 2×10^5^ or 6×10^5^ cells/well) as feeder cells in 24-well plates. Culture medium (complete IMDM with rIL-2) was changed every 2 to 3 days.

### Flow Cytometric Analysis of Cell Surface Antigen Expression

To characterize human peripheral blood, PBMC were labeled with FITC-conjugated αCD3, PC7-conjugated αCD8, PE-conjugated αCD161 and PC5-conjugated αCD56 followed by FACS analysis with a BD FACSCanto II (Figure 1). To analyze the cell surface antigen expression profile of CD3^+^CD8^+^CD161^−^CD56^+^ cells, PBMC were labeled with Brilliant Violet 605™-conjugated αCD3, Pacific Blue™-conjugated αCD8, PC5-conjugated αCD56, Alexa Fluor® 647-conjugated αCD161, PC7-conjugated αCD45RA, FITC-conjugated αCD27, αCD127 or αCD94 and PE-conjugated αCD25, αCD45RO, αCD62L, αCD69 or αNKG2a followed by FACS analysis with a BD LSR II flow cytometer (Figure 2 and 3). To analyze the cell surface antigen expression profile of isolated CD8 T cell subsets after PHA stimulation, cells were labeled with FITC-conjugated αCD3, PC7-conjugated αCD8, PC5-conjugated αCD56 and PE-conjugated αCD27, αCD45RA, αCD45RO, αCD62L, αCD94, αCD161, αNKG2a or αVα24 followed by FACS analysis with a BD FACSCanto II (Figure 5).

### Isolation of CD8 Treg from PBMC

PBMC were labeled with FITC-conjugated αCD3, PC7-conjugated αCD8, PE-conjugated αCD161 and PC5-conjugated αCD56. CD3^+^CD8^+^CD161^−^CD56^+^ (CD56^pos^) and CD3^+^CD8^+^CD161^−^CD56^−^ (CD56^neg^) cells were isolated with a fluorescence activated cell sorter (BD FACSAria™). An aliquot of isolated CD56^pos^ cells was stained with PE-conjugated αCD8β or αVα24 followed by FACS analysis with a BD FACSCanto II (Figure 4B). Isolated CD56^pos^ and CD56^neg^ cells were stimulated with 1.2 μg/ml PHA in complete IMDM with human AB serum in the presence of feeder cells and rIL-2 (100 u/ml) in 24-well plates as described above. Culture medium (complete IMDM with rIL-2) was changed every 2 to 3 days, or when media turned yellow. Cells were cultured for 7–15 days before being analyzed for suppressive activity.

### Wright-Giemsa Stain

Cells isolated from PBMC were suspended in complete IMDM at a concentration of 1×10^5^ cells/ml or lower. Cells (100 μl per slide) were mounted onto slides through cytospin. After drying overnight, cells were stained with Wright-Giemsa reagents. Staining was visualized using a bright-field microscope (Leica DC300).

### CD8 Treg Suppressive Activity Assay (CytoTx Flow assay)

Assays were performed and interpreted as described previously [22]. Briefly, CD4 T cells (target) and CD8 T cells (effector) were centrifuged through Ficoll-Hypaque to remove cell debris. Both target and effector cells were labeled with 3,3′ dihexyloxacarbocyanine (DiOC_6_) (Molecular Probe) (1.5 nM) for 10 min at 37°C. After being washed with IMDM, target cells were labeled with PC-7-conjugated anti-CD4 antibodies for 20 min on ice. Both target and effector cells were resuspended in IMDM supplemented with 10% FBS and antibiotics. Target cells (1–3×10^6^/ml) were then incubated for 3 hr at 37°C in the presence or absence (as control) of αCD3 IgE (1 μg/ml) in a tissue culture incubator. In some experiments, purified mouse IgE antibody (1 μg/ml) was used as isotype control for αCD3 IgE to treat target cells. After extensive washing, target cells (1–3×10^5^) were mixed with effector cells in a U-bottomed 96-well plate at various E/T ratios. After 4 hr of incubation at 37°C under 5% CO_2_, cells were harvested and analyzed by flow cytometry. Percent cytotoxicity was calculated as follows: 100×(% of total apoptotic target cells – % of spontaneous apoptotic target cells)/(100 − % of spontaneous apoptotic target cells).

### Statistical Analysis

Data are expressed as mean±SD. Differences between groups were tested via unpaired two-tailed Student’s t-test.

### Ethics Statement

The use of normal human cells for in vitro experiments was approved by the institutional review boards of the Dana-Farber/Harvard Cancer Center and the Brigham and Women’s Hospital.

## References

[1] Shevach EM (2006) From vanilla to 28 flavors: multiple varieties of T regulatory cells. Immunity 25: 195–201. 16920638 10.1016/j.immuni.2006.08.003

[2] Schwartz RH (2005) Natural regulatory T cells and self-tolerance. Nat Immunol 6: 327–330. 15785757 10.1038/ni1184

[3] Sakaguchi S (2005) Naturally arising Foxp3-expressing CD25+CD4+ regulatory T cells in immunological tolerance to self and non-self. Nat Immunol 6: 345–352. 15785760 10.1038/ni1178

[4] Jiang H, Chess L (2006) Regulation of immune responses by T cells. N Engl J Med 354: 1166–1176. 16540617 10.1056/NEJMra055446

[5] Zou W (2006) Regulatory T cells, tumour immunity and immunotherapy. Nat Rev Immunol 6: 295–307. 16557261 10.1038/nri1806

[6] Roncarolo MG, Battaglia M (2007) Regulatory T-cell immunotherapy for tolerance to self antigens and alloantigens in humans. Nat Rev Immunol 7: 585–598. 17653126 10.1038/nri2138

[7] Sakaguchi S, Yamaguchi T, Nomura T, Ono M (2008) Regulatory T cells and immune tolerance. Cell 133: 775–787. 18510923 10.1016/j.cell.2008.05.009

[8] Jiang H, Zhang SI, Pernis B (1992) Role of CD8+ T cells in murine experimental allergic encephalomyelitis. Science 256: 1213–1215. 1375398 10.1126/science.256.5060.1213

[9] Koh DR, Fung-Leung WP, Ho A, Gray D, Acha-Orbea H, et al (1992) Less mortality but more relapses in experimental allergic encephalomyelitis in CD8−/− mice. Science 256: 1210–1213. 1589800 10.1126/science.256.5060.1210

[10] Rifa’i M, Kawamoto Y, Nakashima I, Suzuki H (2004) Essential roles of CD8+CD122+ regulatory T cells in the maintenance of T cell homeostasis. J Exp Med 200: 1123–1134. 15520244 10.1084/jem.20040395 PMC2211869

[11] Hu D, Ikizawa K, Lu L, Sanchirico ME, Shinohara ML, et al (2004) Analysis of regulatory CD8 T cells in Qa-1-deficient mice. Nat Immunol 5: 516–523. 15098030 10.1038/ni1063

[12] Lu L, Kim HJ, Werneck MB, Cantor H (2008) Regulation of CD8+ regulatory T cells: Interruption of the NKG2A-Qa-1 interaction allows robust suppressive activity and resolution of autoimmune disease. Proc Natl Acad Sci U S A 105: 19420–19425. 19047627 10.1073/pnas.0810383105 PMC2614776

[13] Kim HJ, Verbinnen B, Tang X, Lu L, Cantor H (2010) Inhibition of follicular T-helper cells by CD8(+) regulatory T cells is essential for self tolerance. Nature 467: 328–332. 20844537 10.1038/nature09370 PMC3395240

[14] Correale J, Villa A (2008) Isolation and characterization of CD8+ regulatory T cells in multiple sclerosis. J Neuroimmunol 195: 121–134. 18234356 10.1016/j.jneuroim.2007.12.004

[15] Wei S, Kryczek I, Zou L, Daniel B, Cheng P, et al (2005) Plasmacytoid dendritic cells induce CD8+ regulatory T cells in human ovarian carcinoma. Cancer Res 65: 5020–5026. 15958543 10.1158/0008-5472.CAN-04-4043

[16] Elrefaei M, Ventura FL, Baker CA, Clark R, Bangsberg DR, et al (2007) HIV-specific IL-10-positive CD8+ T cells suppress cytolysis and IL-2 production by CD8+ T cells. J Immunol 178: 3265–3271. 17312176 10.4049/jimmunol.178.5.3265

[17] Uss E, Rowshani AT, Hooibrink B, Lardy NM, van Lier RA, et al (2006) CD103 is a marker for alloantigen-induced regulatory CD8+ T cells. J Immunol 177: 2775–2783. 16920912 10.4049/jimmunol.177.5.2775

[18] Mahic M, Henjum K, Yaqub S, Bjornbeth BA, Torgersen KM, et al (2008) Generation of highly suppressive adaptive CD8(+)CD25(+)FOXP3(+) regulatory T cells by continuous antigen stimulation. Eur J Immunol 38: 640–646. 18266270 10.1002/eji.200737529

[19] Chang CC, Ciubotariu R, Manavalan JS, Yuan J, Colovai AI, et al (2002) Tolerization of dendritic cells by T(S) cells: the crucial role of inhibitory receptors ILT3 and ILT4. Nat Immunol 3: 237–243. 11875462 10.1038/ni760

[20] Jiang H, Ware R, Stall A, Flaherty L, Chess L, et al (1995) Murine CD8+ T cells that specifically delete autologous CD4+ T cells expressing V beta 8 TCR: a role of the Qa-1 molecule. Immunity 2: 185–194. 7895175 10.1016/s1074-7613(95)80079-4

[21] Bisikirska B, Colgan J, Luban J, Bluestone JA, Herold KC (2005) TCR stimulation with modified anti-CD3 mAb expands CD8+ T cell population and induces CD8+CD25+ Tregs. J Clin Invest 115: 2904–2913. 16167085 10.1172/JCI23961 PMC1201661

[22] Hu D, Liu X, Zeng W, Weiner HL, Ritz J (2012) A clonal model for human CD8+ regulatory T cells: unrestricted contact-dependent killing of activated CD4+ T cells. Eur J Immunol 42: 69–79. 22002875 10.1002/eji.201141618 PMC3251657

[23] Pandiyan P, Zheng L, Ishihara S, Reed J, Lenardo MJ (2007) CD4(+)CD25(+)Foxp3(+) regulatory T cells induce cytokine deprivation-mediated apoptosis of effector CD4(+) T cells. Nat Immunol 8: 1353–1362. 17982458 10.1038/ni1536

[24] Sakaguchi S, Powrie F (2007) Emerging challenges in regulatory T cell function and biology. Science 317: 627–629. 17673654 10.1126/science.1142331

[25] Griffin JD, Hercend T, Beveridge R, Schlossman SF (1983) Characterization of an antigen expressed by human natural killer cells. J Immunol 130: 2947–2951. 6574190

[26] Hercend T, Griffin JD, Bensussan A, Schmidt RE, Edson MA, et al (1985) Generation of monoclonal antibodies to a human natural killer clone. Characterization of two natural killer-associated antigens, NKH1A and NKH2, expressed on subsets of large granular lymphocytes. J Clin Invest 75: 932–943. 3884668 10.1172/JCI111794 PMC423627

[27] Kronenberg M (2005) Toward an understanding of NKT cell biology: progress and paradoxes. Annu Rev Immunol 23: 877–900. 15771592 10.1146/annurev.immunol.23.021704.115742

[28] Bendelac A, Savage PB, Teyton L (2007) The biology of NKT cells. Annu Rev Immunol 25: 297–336. 17150027 10.1146/annurev.immunol.25.022106.141711

[29] Loza MJ, Metelitsa LS, Perussia B (2002) NKT and T cells: coordinate regulation of NK-like phenotype and cytokine production. Eur J Immunol 32: 3453–3462. 12442327 10.1002/1521-4141(200212)32:12<3453::AID-IMMU3453>3.0.CO;2-D

[30] Hintzen RQ, de Jong R, Lens SM, Brouwer M, Baars P, et al (1993) Regulation of CD27 expression on subsets of mature T-lymphocytes. J Immunol 151: 2426–2435. 7689607

[31] Lanier LL, Chang C, Phillips JH (1994) Human NKR-P1A. A disulfide-linked homodimer of the C-type lectin superfamily expressed by a subset of NK and T lymphocytes. J Immunol 153: 2417–2428. 8077657

[32] Baume DM, Caligiuri MA, Manley TJ, Daley JF, Ritz J (1990) Differential expression of CD8 alpha and CD8 beta associated with MHC-restricted and non-MHC-restricted cytolytic effector cells. Cell Immunol 131: 352–365. 2122925 10.1016/0008-8749(90)90260-x

[33] Ballas ZK, Rasmussen W (1990) NK1.1+ thymocytes. Adult murine CD4−, CD8− thymocytes contain an NK1.1+, CD3+, CD5hi, CD44hi, TCR-V beta 8+ subset. J Immunol 145: 1039–1045. 1696293

[34] Ritz J, Campen TJ, Schmidt RE, Royer HD, Hercend T, et al (1985) Analysis of T-cell receptor gene rearrangement and expression in human natural killer clones. Science 228: 1540–1543. 2409597 10.1126/science.2409597

[35] Schmidt RE, Murray C, Daley JF, Schlossman SF, Ritz J (1986) A subset of natural killer cells in peripheral blood displays a mature T cell phenotype. J Exp Med 164: 351–356. 3088199 10.1084/jem.164.1.351 PMC2188196

[36] Halin C, Mora JR, Sumen C, von Andrian UH (2005) In vivo imaging of lymphocyte trafficking. Annu Rev Cell Dev Biol 21: 581–603. 16212508 10.1146/annurev.cellbio.21.122303.133159

[37] Alves NL, van Leeuwen EM, Remmerswaal EB, Vrisekoop N, Tesselaar K, et al (2007) A new subset of human naive CD8+ T cells defined by low expression of IL-7R alpha. J Immunol 179: 221–228. 17579041 10.4049/jimmunol.179.1.221

[38] Carrasco J, Godelaine D, Van Pel A, Boon T, van der Bruggen P (2006) CD45RA on human CD8 T cells is sensitive to the time elapsed since the last antigenic stimulation. Blood 108: 2897–2905. 16857986 10.1182/blood-2005-11-007237

[39] Borst J, Hendriks J, Xiao Y (2005) CD27 and CD70 in T cell and B cell activation. Curr Opin Immunol 17: 275–281. 15886117 10.1016/j.coi.2005.04.004

[40] Nolte MA, van Olffen RW, van Gisbergen KP, van Lier RA (2009) Timing and tuning of CD27–CD70 interactions: the impact of signal strength in setting the balance between adaptive responses and immunopathology. Immunol Rev 229: 216–231. 19426224 10.1111/j.1600-065X.2009.00774.x

[41] Tomiyama H, Takata H, Matsuda T, Takiguchi M (2004) Phenotypic classification of human CD8+ T cells reflecting their function: inverse correlation between quantitative expression of CD27 and cytotoxic effector function. Eur J Immunol 34: 999–1010. 15048710 10.1002/eji.200324478

[42] Vossen MT, Matmati M, Hertoghs KM, Baars PA, Gent MR, et al (2008) CD27 defines phenotypically and functionally different human NK cell subsets. J Immunol 180: 3739–3745. 18322179 10.4049/jimmunol.180.6.3739

